# A type III effectiveness-implementation hybrid evaluation of a multicomponent patient navigation strategy for advanced-stage Kaposi’s sarcoma: protocol

**DOI:** 10.1186/s43058-022-00281-7

**Published:** 2022-05-13

**Authors:** Sigrid Collier, Aggrey Semeere, Helen Byakwaga, Miriam Laker-Oketta, Linda Chemtai, Anjuli D. Wagner, Ingrid V. Bassett, Kara Wools-Kaloustian, Toby Maurer, Jeffrey Martin, Samson Kiprono, Esther E. Freeman

**Affiliations:** 1 University of Washington, Seattle, WA USA; 2 Infectious Disease Institute, Kampala, Uganda; 3 Academic Model Providing Access to Healthcare (AMPATH), Eldoret, Kenya; 4 Massachusetts General Hospital, Harvard Medical School, Boston, MA USA; 5 Indiana University, Indianapolis, IN USA; 6 University of California San Francisco, San Francisco, California USA; 7 Moi University, School of Medicine, Department of Internal Medicine, Eldoret, California USA

**Keywords:** Effectiveness-implementation hybrid, Kaposi’s sarcoma, HIV-associated malignancies, Low- and middle-income countries

## Abstract

**Background:**

For people with advanced-stage Kaposi’s sarcoma (KS), a common HIV-associated malignancy in sub-Saharan Africa, mortality is estimated to be 45% within 2 years after KS diagnosis, despite increasingly wide-spread availability of antiretroviral therapy and chemotherapy. For advanced-stage KS, chemotherapy in addition to antiretroviral therapy improves outcomes and saves lives, but currently, only ~50% of people with KS in western Kenya who have an indication for chemotherapy actually receive it. This protocol describes the evaluation of a multicomponent patient navigation strategy that addresses common barriers to service penetration of and fidelity to evidence-based chemotherapy among people with advanced-stage KS in Kenya.

**Methods:**

This is a hybrid type III effectiveness-implementation study using a non-randomized, pre- post-design nested within a longitudinal cohort. We will compare the delivery of evidence-based chemotherapy for advanced-stage KS during the period before (2016–2020) to the period after (2021–2024), the rollout of a multicomponent patient navigation strategy. The multicomponent patient navigation strategy was developed in a systematic process to address key determinants of service penetration of and fidelity to chemotherapy in western Kenya and includes (1) physical navigation and care coordination, (2) video-based education, (3) travel stipend, (4) health insurance enrollment assistance, (5) health insurance stipend, and (6) peer mentorship. We will compare the pre-navigation period to the post-navigation period to assess the impact of this multicomponent patient navigation strategy on (1) implementation outcomes: service penetration (chemotherapy initiation) and fidelity (chemotherapy completion) and (2) service and client outcomes: timeliness of cancer care, mortality, quality of life, stigma, and social support. We will also describe the implementation process and the determinants of implementation success for the multicomponent patient navigation strategy.

**Discussion:**

This study addresses an urgent need for effective implementation strategies to improve the initiation and completion of evidence-based chemotherapy in advanced-stage KS. By using a clearly specified, theory-based implementation strategy and validated frameworks, this study will contribute to a more comprehensive understanding of how to improve cancer treatment in advanced-stage KS.

**Supplementary Information:**

The online version contains supplementary material available at 10.1186/s43058-022-00281-7.

Contributions to the literature

- For advanced-stage Kaposi’s sarcoma, chemotherapy in addition to antiretroviral therapy improves outcomes and saves lives.
- Despite the evidence for chemotherapy, currently only approximately 50% of people with advanced-stage Kaposi’s sarcoma in western Kenya who should receive chemotherapy actually receive it.
- This study will contribute to our understanding of whether a multicomponent patient navigation strategy is effective in improving chemotherapy service penetration (chemotherapy initiation) and fidelity (chemotherapy completion) for advanced-stage Kaposi’s sarcoma in sub-Saharan Africa.

## Background

Kaposi’s sarcoma (KS) continues to be one of the most common HIV-associated malignancies in sub-Saharan Africa, and unfortunately, KS also continues to be deadly [1]. Mortality is estimated to be 45% within 2 years after KS diagnosis, despite improvements in HIV care and more widespread availability of antiretroviral therapy and chemotherapy [1, 2]. For people with advanced-stage KS, chemotherapy in addition to antiretroviral therapy reduces morbidity and mortality [3–6]. Specifically, the combination of antiretroviral therapy and chemotherapy improves KS response rates, the proportion of people with a reduction in measurable tumor burden, by approximately 20–40% as compared to antiretroviral therapy alone [3–6]. Unfortunately, currently up to 50% of people with advanced-stage KS who have an indication for chemotherapy do not initiate treatment with chemotherapy in western Kenya [7].

The reasons for suboptimal chemotherapy initiation and completion for advanced-stage KS are multifactorial including individual (e.g., knowledge of KS, chemotherapy, and cancer), inter-personal (e.g., stigma and lack of social support), community/social (e.g., fatalism about cancer), and environmental factors (e.g., expensive, time-consuming, and complex healthcare systems) [7, 8]. Together these factors coalesce and contribute to suboptimal cancer care for KS.

Patient navigation is a community-based strategy [9] that promotes access to timely diagnosis and treatment of cancer and other chronic diseases, which was designed to overcome resource limitations and barriers to care experienced by marginalized populations. The core component of patient navigation is the patient navigator who addresses peoples’ barriers to care, educates people on their health condition, and regularly contacts people about their treatment status [9, 10]. Patient navigation addresses multiple levels of barriers to care, including key environmental and structural barriers. Financial barriers are often also addressed by patient navigation through financial stipends, which may be a core component of this implementation strategy in resource-limited settings [10, 11]. Patient navigation has been widely adopted to improve HIV care and less frequently cancer care around the globe because it is uniquely well-suited to resource-limited settings [12, 13].

In high-income countries, patient navigation has been shown to be effective and cost-effective for improving outcomes across the cancer care continuum, including cancer screening rates [12]. It also improves the quality of life among people with cancer [12] and may impact social support, coping, and stigma [14–16]. In low- and middle-income countries, there is early evidence for the benefits of patient navigation to promote cancer screening, including breast cancer screening in Kenya [15, 17]. To date, fewer studies have focused on evaluating whether patient navigation improves cancer treatment in low- and middle-income countries.

Based on our work describing major barriers to accessing KS care [8] and the evidence for patient navigation in oncology and HIV care, our institutional partners at the Academic Model Providing Access to Health (AMPATH) program in Kenya decided to implement a multicomponent patient navigation strategy as part of their KS Center of Excellence to improve initiation (service penetration) and completion (fidelity) of evidence-based chemotherapy among people with advanced-stage KS (Table 1). This represents a unique opportunity to evaluate the impact of a multicomponent patient navigation strategy when it is implemented as part of routine care in a sub-Saharan African setting.

**Table 1:** Components of the implementation strategy

			Multicomponent Patient Navigation Strategy		
Component	Education	Physical navigation	Transportation stipend	Health insurance enrollment stipend	Peer mentor
**Justification**	Lack of knowledge about KS treatment, side effects, and beliefs about the outcome of treatment (limb amputation) were barriers to KS treatment in qualitative study	Encouragement and reminders by healthcare workers were identified in qualitative study as facilitators of KS treatment	Cost of transportation was identified in qualitative study as a barrier to KS treatment	Cost of chemotherapy and health insurance was identified in qualitative study as a barrier to KS treatment	Lack of social support and belief that everyone with cancer (KS) dies were barriers to KS treatment, knowing a KS survivor was a facilitator in qualitative study
**Mechanism of action (COM-B)**	Capability-psychological Motivation-reflective	Opportunity-physical Capability-psychological	Opportunity-physical	Opportunity-physical	Motivation-automatic Opportunity-social
**COM-B intervention function targeted**	Education and modelling	Environmental restructuring, training, and enablement	Enablement	Enablement	Modelling and enablement
**Definition**	Increasing knowledge or understanding	An individual who identifies a person’s barriers to the diagnosis and treatment of a disease and helps the person overcome them.	Financial stipend to offset cost of transportation	Financial stipend to offset cost of health insurance	An individual with a history of the same disease who provides support during the treatment process
**Actor(s)**	Patient navigator	Patient navigator	Patient navigator or coordinator	Patient navigator or coordinator	KS survivor
**Action(s)**	1) Video about KS etiology and disease course 2) Video about KS treatment	1) Physically navigate to visits 2) Connect to services 3) Reminder about health insurance registration 4) Reminder about clinic visits	1) Provide stipend to offset the cost of transportation to first oncology visit and all chemotherapy visits (x6)	1) Provide stipend for 1-year fees for health insurance	Speak to person with KS on the phone or in-person about: (1) their experience (2) guidance on next steps (3) encouragement to continue KS treatment (4) Refer to patient navigator when services are needed
**Target(s) of action**	Person with KS	Person with KS	Person with KS	Person with KS	Person with KS
**Temporality**	(1) At biopsy result visit (2) At first oncology visit	(1) First oncology visit, first chemotherapy (2) Biopsy Result Visit or First Oncology Visit (3) At least once every 2 weeks for each person with KS not yet registered (4) For first oncology visit, every chemotherapy x 6	(1) At each of the above visits	(1) At time of complete health insurance registration	(1) After first oncology visit and after each chemotherapy
**Dose**	(1)1 occurrence (10 min) (2) 1 occurrence (15 min)	(1) 2 occurrences (2) at least 1 occurrence (3) at least 1 occurrence (4) at least 7 occurrences	(1) 7 occurrences	(1) 1 occurrence	(1) at least 7 occurrences (at least 15 min each occurrence)

This study addresses an urgent need for evidence-based strategies to improve service penetration of and fidelity to cancer treatment in KS, which continues to be a common, deadly, and debilitating disease in sub-Saharan Africa. It also incorporates the strength of a type III effectiveness-implementation hybrid study that evaluates primary implementation outcomes as well as secondary clinical effectiveness outcomes, thus potentially accelerating the process of implementing evidence-based interventions into routine practice [18].

### Implementation outcomes terminology

**Fig. 1:**
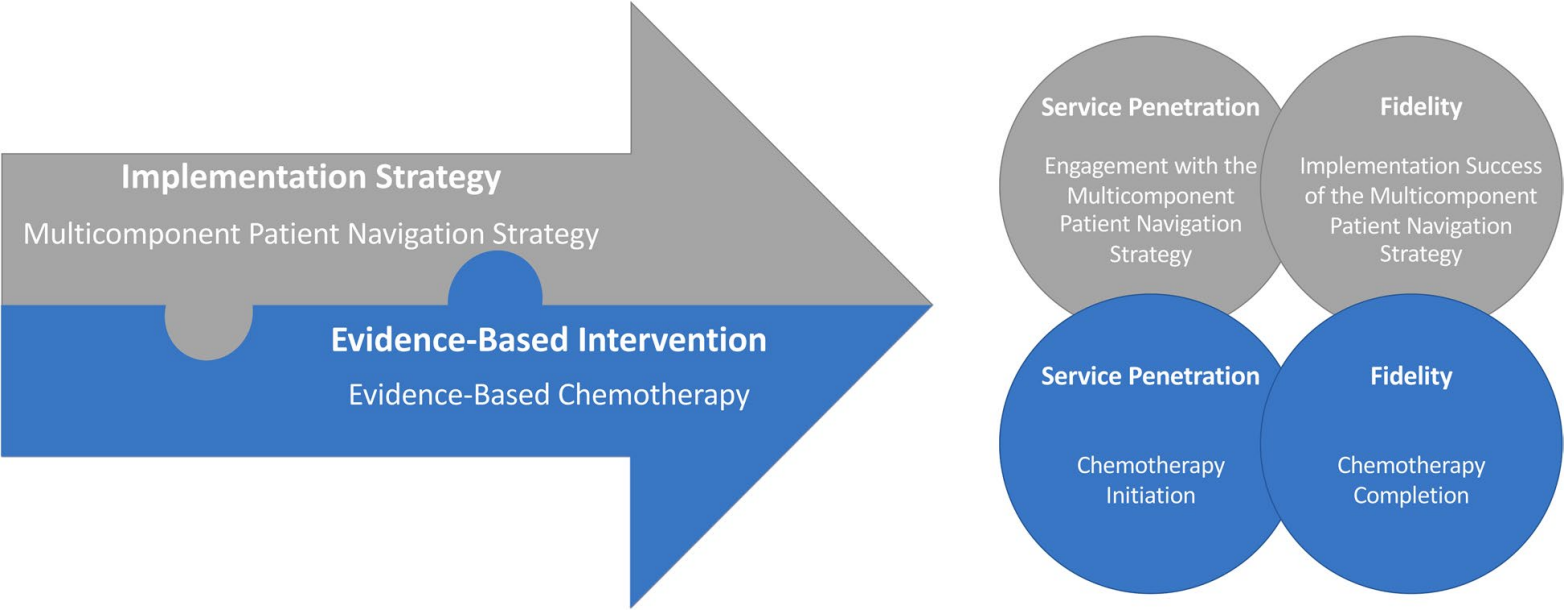
Implementation science terminology: operationalization of implementation outcomes

Throughout this protocol, we use Proctor et al.’s taxonomy of implementation outcomes as the framework to define and distinguish implementation, service, and client outcomes [19]. We also distinguish two different levels of fidelity and service penetration corresponding to (1) the evidence-based intervention, which is chemotherapy for advanced-stage KS and (2) the implementation strategy, which is a multicomponent patient navigation strategy (Fig. 1).

The following illustrates this distinction for service penetration, which Proctor et al. define as “the number of eligible persons who use a service, divided by the total number of persons eligible for the service.” [19] (1) Service penetration for the evidence-based intervention (chemotherapy) could also be called chemotherapy initiation (Table 2). (2) Service penetration for the implementation strategy (the multicomponent patient navigation strategy) could also be called engagement with the multicomponent patient navigation strategy (Table 3). Likewise, fidelity to the evidence-based intervention among people with KS could be called chemotherapy completion, while fidelity to the implementation strategy at AMPATH could be called the implementation success for the multicomponent patient navigation strategy.

**Table 2:** Study outcomes for the evidence-based intervention

**Implementation science terminology**	**Definition**		**Study terminology**	
**Evidence-based intervention**	Evidence-based interventions are supported by research that has established a causal relationship between the intervention and a specified improvement in individual- or population-level health behaviors, health outcomes, or health-related environments [20].		Evidence-based chemotherapy for advanced-stage Kaposi’s sarcoma	
**Primary implementation outcomes - intervention**				
Implementation science terminology	**Definition**	**Study terminology**	Level of evaluation	Outcome measures
Service Penetration	The number of eligible persons who use a service, divided by the total number of persons eligible for the service [19].	Chemotherapy initiation	Individual level (person with KS)	Cumulative incidence of receiving the first dose of chemotherapy after KS diagnosis accounting for death as a competing event.
Fidelity	The degree to which an intervention was implemented as it was prescribed in the original protocol or as it was intended by the program developers [19].	Chemotherapy completion	Individual level (person with KS)	Cumulative incidence of chemotherapy completion, defined as completing >6 cycles of chemotherapy, completing all cycles recommended by the provider (occasionally <6), or the person with KS completing the prescribed number of cycles up until a provider-driven regimen change, accounting for death as a competing event.
**Secondary service outcomes—intervention**				
Timeliness	The reduction of waits and harmful delays for both those who receive and those who give care [21].	Time to oncology consultation	Individual level (person with KS)	Time in days from when the biopsy result is given to the person with KS to the first oncology consultation
**Secondary client and health outcomes—intervention**				
		Mortality	Individual level (person with KS)	Cumulative incidence of death
		Quality of Life	Individual level (person with KS)	Total Score Quality of Life (Medical Outcomes Study HIV Health Study) [22]
		Stigma	Individual level (person with KS)	Total Score Stigma (Abridged Berger HIV Stigma Scale) [23]
		Social Support	Individual level (person with KS)	Total Score Social Support (MSPSS) [22]

**Table 3:** Study outcomes for the implementation strategy (multicomponent patient navigation strategy)

**Implementation science terminology**	**Definition**		**Study terminology**	
**Implementation strategy**	Implementation strategies are the specific means or methods for adopting and sustaining evidence-based interventions [24]		Multicomponent patient navigation strategy	
**Implementation outcomes—implementation strategy**				
**Implementation science terminology**	**Definition**	**Study terminology**	**Level of evaluation**	**Outcome measures**
Service penetration	The “number of eligible persons who use a service, divided by the total number of persons eligible for the service.” [19]	Engagement with the multicomponent patient navigation strategy	Individual level (person with KS)	Proportion of clients who qualify for patient navigation who have at least one contact with a patient navigator or peer mentor within 90 days after receiving a KS diagnosis.
Fidelity	The “degree to which an implementation strategy was implemented as it was prescribed in the original protocol or as it was intended by the program developers.” [19]	Implementation success for the multicomponent patient navigation strategy	Individual level (person with KS)	Proportion of clients who received all 6 components of the multicomponent patient navigation strategy as designed (described in detail in Table 1) among clients engaged in patient navigation (see service penetration)
Fidelity (dose)	A component of fidelity: “The amount of program delivered.” [19]	Dose of physical navigation and care coordination	Individual level (person with KS)	Total number of interactions with the patient navigator within the first year among clients engaged in patient navigation (see service penetration)
		Dose of peer mentorship		Total number of interactions with the peer mentor within the first year among clients engaged in patient navigation (see service penetration)
		Dose of educational videos		Total number of viewed videos within the first year among clients engaged in patient navigation (see service penetration)
Acceptability	“The perception among implementation stakeholders that a given treatment, service, practice, or innovation is agreeable, palatable, or satisfactory.” [19]	Acceptability of the multicomponent patient navigation strategy	Individual level (person with KS) Patient Navigators Nurses Physicians Administrators	Total scores of acceptability of multicomponent patient navigation strategy on questionnaire [25] Semi-structured interview
		Acceptability of peer mentorship	Individual level (person with KS) Peer mentors	Total scores of acceptability of peer mentorship on questionnaire [25] Semi-structured interview
		Acceptability of the health insurance stipend	Individual level (Person with KS)	Total scores of acceptability of health insurance stipend on questionnaire [25] Semi-structured Interview
		Acceptability of the travel stipend	Individual level (Person with KS)	Total scores of acceptability of travel stipend on questionnaire [25] Semi-structured interview
Feasibility	“The extent to which a new treatment, or an innovation, can be successfully used or carried out within a given agency or setting.” [19]	Feasibility of the multicomponent patient navigation strategy	Person with KS Navigators Peer mentors Nurses Physicians Administrators	Total scores of feasibility of multicomponent patient navigation strategy on questionnaire [25] Semi-structured Interview
		Feasibility of peer mentorship	Peer Mentors	Total scores of feasibility of peer mentorship on questionnaire [25] Semi-structured Interview
		Feasibility of the health insurance stipend	Individual level (person with KS)	Total scores of feasibility of health insurance stipend on questionnaire [25] Semi-structured Interview
		Feasibility of the travel stipend	Individual level (Person with KS)	Total scores of feasibility of travel stipend on questionnaire [25] Semi-structured Interview
Appropriateness	The “perceived fit, relevance, or compatibility of the innovation or evidence-based practice for a given practice setting, provider, or consumer; and/or perceived fit of the innovation to address a particular issue or problem.” [19]	Appropriateness of the multicomponent patient navigation strategy	Individual level (Person with KS) Patient Navigators Peer mentors Nurses Physicians Administrators	Total scores of appropriateness of multicomponent patient navigation on questionnaire [25] Semi-structured interview
		Appropriateness of peer mentorship	Individual level (person with KS) Peer mentors	Total scores of appropriateness of peer mentorship on questionnaire [25] Semi-structured interview
		Appropriateness of the health insurance stipend	Individual level (person with KS)	Total scores of appropriateness of health insurance stipend on questionnaire [25] Semi-structured interview
		Appropriateness of the travel stipend	Individual level (person with KS)	Total scores of appropriateness of travel stipend on questionnaire [25] Semi-structured interview
**Client outcomes—implementation strategy**				
Satisfaction	Satisfaction with “the general service experience, including such features as waiting times, scheduling, and office environment.” [19]	Satisfaction with cancer care	Individual level (person with KS)	Total scores of patient satisfaction with cancer care (PSCC) [26]
		Satisfaction with the patient navigator	Individual level (person with KS)	Total scores of patient satisfaction with navigator interpersonal relationship with navigator (PSN-I) [27, 28]
		Satisfaction with the per navigator	Individual level (person with KS)	Total scores of patient satisfaction interpersonal relationship with peer mentor (adapted PSN-I) [27, 28]

### Objectives

1. Evaluate the impact of a multicomponent patient navigation strategy on service penetration of, and fidelity to, evidence-based chemotherapy for people with advanced-stage KS by comparing the pre-navigation period (2016–2020) to the post-navigation period (2021–2024). We hypothesize that the multicomponent patient navigation strategy will increase service penetration of and fidelity to evidence-based chemotherapy post-navigation as compared to pre-navigation among people with advanced-stage HIV-associated KS.
2. Evaluate the impact of a multicomponent patient navigation strategy on (a) service outcomes, including timeliness (time to oncology consultation), and (b) client outcomes, including mortality, quality of life, stigma, and social support. We hypothesize that a multicomponent patient navigation strategy will decrease time to oncology consultation, decrease KS-associated mortality, increase the quality of life, decrease stigma, and increase social support post-navigation as compared to pre-navigation among people with advanced-stage HIV-associated KS.
3. Describe the implementation process and degree to which the multicomponent patient navigation strategy was successfully implemented, focused on service penetration, fidelity, acceptability, feasibility, appropriateness, and client satisfaction.
4. Identify the determinants of successful implementation of the multicomponent patient navigation strategy, specifically focused on determinants of service penetration of and fidelity to the multicomponent patient navigation strategy.

## Methods

### Overview/study design

This study is a hybrid effectiveness-implementation type III study, [18] using a non-randomized, pre- and post-design nested within a single-center longitudinal cohort. Our control group is newly diagnosed people with KS who were enrolled in this longitudinal cohort study during the pre-navigation period (2016–2020), and our study group is newly diagnosed people with KS who are enrolled in the post-navigation period (2021–2024).

### Study setting and population

This study will take place at the Academic Model Providing Access to Health (AMPATH) in Eldoret, Kenya. AMPATH is an academic medical partnership between Moi University School of Medicine, Moi Teaching and Referral Hospital and several North American and European Universities led by Indiana University. AMPATH oversees over 50 HIV primary care clinics and delivers care to ~160,000 clients in western Kenya. Dermatology and Oncology services including diagnostic biopsy and treatment services for KS are available within the AMPATH healthcare system. As part of routine clinical care, the KS Center for Excellence at AMPATH implemented a multicomponent patient navigation strategy in 2021. All clients who are diagnosed with KS are screened using clinical evaluation, AIDS Clinical Trials Group Oncology Committee (ACTG) staging criteria, and WHO staging criteria for KS. AMPATH KS Center of Excellence invites any clients with KS who meet criteria for chemotherapy (based on any of the following: local guidelines, ACTG T1 disease, or WHO criteria for moderate to severe KS) to participate in the multicomponent patient navigation strategy [29].

The parent longitudinal cohort enrolls all clients of any age with newly diagnosed KS. This includes clients with newly diagnosed KS who have either biopsy confirmed KS or are diagnosed with KS on clinical grounds alone when a biopsy is unsafe (e.g., some oral lesions and conjunctival lesions). We will exclude clients who have a prior biopsy confirmed diagnosis of KS or those who are unable to provide consent.

For this study, the control group will include all clients with advanced-stage newly diagnosed KS enrolled in the parent cohort who are eligible for chemotherapy (based on the criteria outlined above) enrolled during the pre-navigation period (2016–2020). The study group will include all clients with advanced-stage newly diagnosed KS enrolled in the parent cohort who are eligible for chemotherapy and are engaged in the multicomponent patient navigation strategy during the post-navigation period (2021–2024).

We will also enroll healthcare workers who are involved in the multicomponent patient navigation strategy and/or the care of clients with KS to understand the impact of the implementation strategy on routine clinical care. This will include physicians, clinical officers, nurses, social workers, patient navigators, peer mentors, and health insurance officers.

### Evidence-based intervention

In HIV-associated KS, treatment with chemotherapy is recommended in addition to antiretroviral therapy based on consensus guidelines for clients with KS with any of the following: symptomatic visceral disease (pulmonary or gastrointestinal), extensive oral KS lesions that interfere with chewing or swallowing, painful or disabling tumors, life-threatening or functionally disabling disease, and progressive or persistent KS despite antiretroviral therapy [30]. The most common chemotherapy regimen in western Kenya is bleomycin-vincristine, followed by the combination of BV and doxorubicin, and the combination of etoposide and gemcitabine [7]. However, this may be changing, as paclitaxel is increasingly incorporated into KS treatment regimens in East Africa based on recent evidence of superior progression free survival compared to bleomycin-vincristine [31]. Since we are evaluating evidence-based chemotherapy as part of routine clinical care for KS, the chemotherapy regimen will be dependent upon local availability and could change in response to local drug supply or guidelines. As such, there may be variation in the efficacy and side-effect profile of the available chemotherapy regimens [31].

### Implementation strategy

The multicomponent patient navigation strategy was designed using intervention mapping, a structured stepwise process for identifying interventions that target key factors associated with a given behavior, as the guiding framework. We used the first three steps of intervention mapping [32] to guide the overarching process as follows: (1) Logic Model of the Problem: Conduct a needs assessment; (2) Logic Model of Change: Identify determinants of chemotherapy initiation and completion; and (3) Program Design: Identify behavior change mechanisms and evidence-based intervention components. As part of Step 3 in Intervention Mapping, we used the Capability, Opportunity, and Motivation Model of Behavior (COM-B) as the unifying behavioral theory, and the Behavior Change Wheel to identify evidence-based strategies corresponding to the key behavioral mechanisms of chemotherapy service penetration and fidelity. Detailed specification of each of the components and the corresponding COM-B theoretical behavioral mechanisms is outlined in Table 1. A brief description of the six primary components of the multicomponent patient navigation strategy is outlined below.

#### Component 1: physical navigation and care coordination

The responsibilities of the patient navigator will include assistance arranging transportation to oncology and chemotherapy visits, meeting clients on arrival to the health center and physically guiding the clients to their first oncology and first chemotherapy appointments, oncology and chemotherapy visit reminders, guiding clients to health insurance enrollment assistance, and connection to other social services based on each patient’s needs.

#### Component 2: education

The educational component will include educational videos viewed at two-time points: the first clinic visit after KS diagnosis and the initial oncology consultation. The first educational video focuses on the etiology of KS, the natural disease course of KS, and KS diagnostic procedures. It was designed to address the key barriers and facilitators of early diagnosis of KS. The second educational video focuses on the treatment of KS including antiretroviral therapy for people with HIV-associated KS, the treatment options for KS, detailed explanations of chemotherapy regimens for KS, and potential side effects from chemotherapy. The educational video on KS treatment was designed to address key barriers and facilitators to chemotherapy initiation and completion. Both educational videos were developed as part of a participatory process, and feature KS survivors, sharing a message of hope for the survival of KS through treatment with chemotherapy. Both videos were found to be acceptable and informative during field testing with KS survivors.

#### Component 3: travel stipend

All clients will receive a stipend to assist with the cost of their transportation for KS-related oncology care and treatment until the completion of their prescribed treatment course.

#### Component 4: assistance with enrollment in health insurance

Clients will be connected by patient navigators to health insurance officers who will assist them in registering for the Kenyan health insurance, the National Hospital Insurance Fund.

#### Component 5: health insurance stipend

The KS Center of Excellence will provide assistance in paying for the entire first year of health insurance for all clients.

#### Component 6: peer mentorship

Clients will be assigned a peer mentor, who is a KS survivor from the same region in western Kenya. Peer mentors will contact the client prior to their first chemotherapy and subsequently after each chemotherapy visit to offer support and when needed connect the client back to a patient navigator or clinical health professionals for additional services or assistance.

### Methods: study measures

#### Primary implementation outcomes: evidence-based chemotherapy (Table 2)

The primary outcomes of service penetration (chemotherapy initiation) and fidelity (chemotherapy completion) are defined in Table 2.

#### Secondary effectiveness outcomes: evidence-based chemotherapy (Table 2)

Secondary outcomes include: timeliness (time from KS diagnosis to oncology consultation), mortality, quality of life (Medical Outcomes Study HIV Health Study) [33, 34], stigma (Berger HIV Stigma Scale) [23, 35], and social support (MSPSS) [36, 37]. These previously validated questionnaires, which have been used in sub-Saharan Africa, will be adapted for use in Kenya including translation to Swahili, back translation to English, and field testing for reliability and content validity.

#### Process evaluation: evaluating the roll-out of the multicomponent patient navigation strategy

In this study, we will also evaluate the implementation process and the degree to which the multicomponent patient navigation strategy was successfully implemented because during routine implementation, and penetration may be variable and modifications (unplanned, reactive adaptations) may compromise fidelity [38]. Additionally, acceptability, feasibility, and appropriateness will be important considerations for future adaptation and implementation of the multicomponent patient navigation strategy.

#### Process outcomes: multicomponent patient navigation strategy (Table 3)

The process outcome measures for describing the implementation process (roll-out) of the multicomponent patient navigation strategy are outlined below.

Service penetration and fidelity: A detailed description of the operationalization of service penetration and fidelity to the multicomponent patient navigation strategy is outlined in Table 3.

Acceptability, feasibility, and appropriateness: We will use quantitative questionnaires evaluating Acceptability of Intervention Measure (AIM), Intervention Appropriateness Measure (IAM), Feasibility of Intervention Measure (FIM) [25], and semi-structured interviews to measure (1) acceptability and appropriateness (AIM, IAM) [25] of the multicomponent patient navigation strategy [19] among clients and (2) the acceptability, feasibility, and appropriateness among patient navigators and peer mentors. We will use a purely quantitative approach with validated measures of acceptability, feasibility, and appropriateness (AIM, IAM, FIM) [25], among other healthcare workers (excluding patient navigators and peer mentors).

Satisfaction: Among all clients, we will use previously validated questionnaires, which have been adapted and field-tested for this study, to evaluate satisfaction with cancer care [26], patient satisfaction with the interpersonal relationship with patient navigator [27, 28], and patient satisfaction with the interpersonal relationship with peer mentor using previously validated questionnaires [27, 28].

#### Implementation determinants, multicomponent patient navigation strategy

Among clients, patient navigators, and peer mentors, we will assess the determinants of service penetration of (engagement with) and fidelity to the multicomponent patient navigation strategy, using the Theoretical Domains Framework (TDF) as a guiding framework [39]. We have developed structured questionnaires and semi-structured interview guides that evaluate key domains from the TDF (Skills, Knowledge, Social Influences, Environmental Context and Resources, Social Role and Identity, Beliefs about Consequences, Goals, Emotion, and Optimism). We will administer structured questionnaires to all enrolled clients and conduct semi-structured interviews with a stratified purposive sample of clients who participated in the multicomponent patient navigation strategy, as well as a subset of eligible clients who did not participate. Among patient navigators and peer mentors, we will also describe the determinants of fidelity to the multicomponent patient navigation strategy, using structured questionnaires and semi-structured interviews focused on key domains of the TDF (Skills, Social/Professional Role and Identity, Beliefs about Consequences, Social Influences, Reinforcement, Behavioral Regulation, Environmental Context and Resources, and Emotion).

#### Data collection and data sources

There are three main data sources: structured questionnaires, semi-structured interviews, and chart review of the electronic medical record. All enrolled clients and a representative sample of patient navigators, peer mentors, and healthcare workers will be asked to complete a series of structured questionnaires (Tables 2, 3, and 4), which will be adapted for use in Kenya. A purposive sample of clients, patient navigators, and peer mentors will also be invited to participate in semi-structured interviews.

**Table 4:** Study timeline

	24 months Pre-PN	PN	Baseline	Month 3	Month 6	Month 9	Month 12	Month 19	Month 24
Demographics	x		x						
**Implementation and service outcomes for evidence-based chemotherapy**									
Oncology care—chart review	x			x	x	x	x	x	x
**Client outcomes**									
Vital status	x			x	x	x	x	x	x
Quality of life (MOS-HIV)	x		x	x	x	x	x		x
Social support	x		x	x	x	x	x		x
Stigma	x		x	x	x	x	x		x
**Process outcomes and patient satisfaction for multicomponent patient navigation strategy**									
Fidelity				x	x	x	x		x
Acceptability				x	x		x		
Feasibility				x	x		x		
Appropriateness				x	x		x		
Service penetration				x	x	x	x		
Satisfaction (PSCC)			x	x	x	x	x		
Satisfaction with Patient navigator				x	x	x	x		
Satisfaction with peer mentor				x	x	x	x		
**Determinants of the implementation of multicomponent patient navigation strategy**									
Client interviews				x	x				
Healthcare worker questionnaires							x		
Patient navigation questionnaires			x	x	x	x	x		

*PN* multicomponent patient navigation strategy

For chart review, we will review medical records at the client’s HIV primary clinic and all relevant oncology clinics. We will collect information on oncology and/or chemotherapy visits, provider seen, and therapy given including dates of (a) initial visit to oncology clinic, (b) initial evaluation by an oncology provider qualified to make a treatment decision, (c) first chemotherapy dose, and (d) the timing and number of subsequent chemotherapy doses. As a part of the longitudinal evaluation, clients will be contacted every 3 months for the first year after enrollment and every 6 months thereafter to evaluate the vital status and any oncology care received outside of the AMPATH health system (Table 4). In the event we lose contact with a client not known to be dead based on chart review, we will initiate tracking his/her vital status in the community [6].

Data collection will be performed by either trained research staff or (where appropriate) collected by patient navigators and peer mentors as part of routine clinical care.

### Methods: analysis

#### Primary implementation outcomes: evidence-based chemotherapy

We will estimate the cumulative incidence of chemotherapy initiation and completion accounting for death as a competing event using the Aalen-Johansen estimator [40]. We will then use a Cox proportional hazards regression model to compare the cumulative incidence of chemotherapy initiation and completion in the pre-navigation period (2016–2020) to the post-navigation period (2021–2024) adjusting for important determinants of chemotherapy initiation and completion (e.g., stage at KS diagnosis, CD4 count, age, sex, and chemotherapy regimen). There will be a 3-month washout period to ensure that the clients enrolled in the post-navigation period experience the multicomponent patient navigation strategy after it is fully implemented.

#### Secondary effectiveness outcomes: evidence-based chemotherapy

We will also use a Cox regression model to compare time to oncology consultation and mortality in the post-navigation period to the pre-navigation period. For other client outcomes (quality of life, stigma, and social support), we will compare the pre-navigation and post-navigation periods using generalized linear regression with a Gaussian family and identity link for continuous outcomes and binomial family with log link for binary outcomes.

#### Process outcomes: multicomponent patient navigation strategy

##### Process outcomes: qualitative

We will use a theory-based, framework approach to qualitative data analysis focused on acceptability, appropriateness, and feasibility of the multicomponent patient navigation strategy, while allowing for emergent themes that do not fit within the pre-defined Proctor implementation outcomes taxonomy [19]. Interviews will be independently coded by two researchers trained in qualitative data analysis, using NVivo qualitative data analysis software, and any discrepancies will be resolved by consensus.

##### Process outcomes: quantitative

We will use descriptive statistics (mean, standard deviation, range) to describe acceptability, appropriateness, and feasibility (AIM, IAM, FIM) [25] among healthcare workers, and acceptability and appropriateness among clients. We will stratify our analysis based on the type of stakeholder (e.g., clients, HIV providers, dermatology providers, oncology providers, and implementation partners) to understand differences in the acceptability, appropriateness, and feasibility (AIM, IAM, FIM) [25] between different stakeholder types. In light of the sample size of healthcare workers (*N*=50), we will not conduct hypothesis testing for differences between these groups, in accordance with best practices.

We will report descriptive statistics (median, standard deviation, median, interquartile range, and range as applicable) for service penetration, fidelity, dose, and patient satisfaction with the implementation strategy (as defined in Table 3). We will explore whether service penetration of, and fidelity to, the multicomponent patient navigation strategy changes the magnitude of the association between exposure to the multicomponent patient navigation strategy and the primary implementation outcomes (service penetration of and fidelity to evidence-based chemotherapy). We will perform stratified analyses by service penetration of (engagement) and fidelity to the multicomponent patient navigation strategy within our adjusted Cox proportional hazards regression model comparing the cumulative incidence of chemotherapy initiation and completion in the post-navigation period to the pre-navigation period.

### Mixed-methods

We will use a convergent design for our mixed-methods evaluation, where we triangulate the quantitative results from structured questionnaires with the qualitative semi-structured interviews. We will integrate quantitative and qualitative results, using qualitative data to provide depth of understanding [41–43] for the analysis of acceptability, appropriateness, and feasibility (AIM, IAM, FIM) of the multicomponent patient navigation strategy among clients (acceptability and appropriateness only), patient navigators, and peer mentors [25].

#### Implementation determinants: multicomponent patient navigation strategy

##### Determinants: qualitative

We will use the Theoretical Domains Framework [44], to define our a priori coding framework in a theory-based, framework approach to this qualitative assessment, while allowing for emergent themes that do not fit within the pre-defined Theoretical Domains Framework [44], using the same analytic methods described above. The qualitative evaluation will include patient navigator and peer mentors’ experiences around the determinants of service penetration of and fidelity to the multicomponent patient navigation strategy. In addition, as part of our evaluation of the determinants of service penetration of the multicomponent patient navigation strategy, we will perform an embedded (stratified) analysis comparing clients who did and did not engage in the multicomponent patient navigation strategy (Table 3). We define engagement as having met with a patient navigator or peer mentor at least once within 90 days after receiving a KS diagnosis.

##### Determinants: quantitative

Our quantitative evaluation will include exploratory bivariate analyses to identify individual-level factors (e.g., age, sex, tribe, socioeconomic status, characteristics, stigma) associated with service penetration of the multicomponent patient navigation strategy, using generalized linear regression with binomial family and a log link for the binary outcome of service penetration (engagement).

#### Mixed-methods

We will use a convergent design for our mixed-methods evaluation, where we triangulate the quantitative results from structured questionnaires with the qualitative semi-structured interviews. We will integrate quantitative and qualitative results, using qualitative data to provide depth of understanding [41–43] for the analysis of (1) determinants of service penetration of the multicomponent patient navigation strategy and (2) determinants of fidelity to the multicomponent patient navigation strategy.

#### Methods: sample size and power

We will recruit all newly diagnosed clients with advanced-stage KS. Between 2016 and 2020, we enrolled 367 clients, and 242 clients met the criteria for advanced KS based on either ACTG T1 or WHO “Severe KS” criteria (pre-navigation; 66%). In 2019, there were 96 clients with newly diagnosed KS within the AMPATH network. Based on this, we estimate that we will enroll 335 clients during the post-navigation time period (3.5 years, 2021–2024). Assuming we enroll 335 clients, we estimate that 221 clients will have advanced-stage KS. This makes a total of 463 clients in the pre-navigation and post-navigation period combined.

#### Primary implementation outcomes, evidence-based chemotherapy, and service penetration and fidelity

For the primary implementation outcomes of service penetration (chemotherapy initiation) and fidelity (chemotherapy completion), we anticipate 242 clients in the pre-navigation period and 221 clients in the post-navigation period. If we assume a type I error of 5% and 56% initiation of chemotherapy by 1 year accounting for death as a competing event, we will have 80% power to detect a relative hazard of 0.70 or greater.

#### Secondary effectiveness outcomes, evidence-based chemotherapy, and timeliness and mortality

Assuming that the estimated proportion of newly diagnosed clients who have an oncology consultation is similar to the proportion who initiate chemotherapy, the estimated power to detect changes in the time to oncology consultation will be similar to the primary outcome (chemotherapy initiation). For the outcome of mortality, during the pre-navigation time period between 2016 and 2020, we observed 141 deaths in the 367 enrolled clients (pre-navigation; 38% at 1 year). If we assume a similar mortality rate in the post-navigation period (2021–2024) and we enroll 335 clients, we are likely to observe around 127 deaths. Thus, assuming a type I error of 5% and a mortality of 38% at 1 year, we will have 80% power to detect a relative hazard of 0.67 or greater when comparing the pre-navigation period to the post-navigation period.

#### Process outcomes and implementation determinants: multicomponent patient navigation strategy

##### Quantitative

In addition to enrolled clients (described above), a total of 50 healthcare workers will participate in structured questionnaires. Healthcare workers will include all patient navigators and all peer mentors as well as a representative sample of health insurance officers, social workers, HIV providers (physicians, nurses, and clinical officers), oncology providers (physicians, nurses, and clinical officers), and dermatology providers (physicians, nurses, and clinical officers).

##### Qualitative

We will perform semi-structured interviews with a subset of clients who are eligible to participate in the multicomponent patient navigation strategy. The sample size will be driven by reaching thematic saturation, which will likely be achieved by interviewing 20 clients who participated in the multicomponent patient navigation strategy and 20 who did not participate [45]. For healthcare worker semi-structured interviews, the sample size will also be driven by reaching thematic saturation [45], which will likely be achieved with a total of 20 patient navigators and peer mentor interviews [44].

## Discussion

In western Kenya, currently less than 50% of people with KS who qualify for chemotherapy receive it [7]. Strategies to improve outcomes in cancer care must address key steps in the cancer care cascade and account for important environmental and structural barriers to care, including transportation, cost, health system complexity, stigma, and social support. This primary goal of this study is to evaluate whether a multicomponent patient navigation strategy increases service penetration of and fidelity to evidence-based chemotherapy for advanced-stage KS.

Patient navigation is well-suited to improving outcomes in HIV-associated malignancies, such as KS in LMICs. This study will build on the evidence for patient navigation for oncology care in resource-limited settings, providing evidence for an implementation strategy that can be adapted to other resource-limited contexts in sub-Saharan Africa and throughout the world. While many studies have focused on patient navigation as a strategy to improve cancer screening rates and satisfaction with cancer care, fewer studies have focused on increasing treatment initiation. There have been other efforts to establish cancer patient navigation in sub-Saharan Africa, though to our knowledge, these have focused primarily on increasing adherence to chemotherapy and do not address barriers prior to the initiation of chemotherapy [46]. In summary, our study is novel in its evaluation of the impact of patient navigation on cancer treatment initiation and completion in sub-Saharan Africa.

This approach is also unique because the hybrid design represents an opportunity to evaluate both relevant implementation outcomes as well as important long-term client and effectiveness outcomes. A recent review found that none of the studies in low-and-middle-income countries evaluating patient navigation reported on relevant long-term clinical outcomes [15], and few studies in high-income countries have specifically evaluated important long-term clinical outcomes, including cancer-related survival [12].

It is also the first study, to our knowledge, to use implementation science frameworks to guide the evaluation of a multicomponent patient navigation strategy for cancer in a low- and middle-income country. Although several published evaluations of patient navigation in low- and middle-income countries reported on common implementation science outcomes, none reported the frameworks or theories used to guide their evaluation [15]. By using well-known rigorous frameworks to guide our evaluation, we are uniquely positioned to generate evidence for patient navigation in cancer care that can be directly compared to similar studies in other contexts.

A limitation of this study is the lack of randomization. Randomization of the multicomponent patient navigation strategy was not felt to be ethical by the KS Center of Excellence at AMPATH because in western Kenya treatment for KS is currently suboptimal and associated with unacceptably high mortality. Additionally, there is a substantial risk of experimental contamination in the control arm with individual or cluster randomization. This is due to high levels of information sharing among individuals within a given oncology clinic, high regional population mobility, and high crossover between clinics in western Kenya. Although causal conclusions may be limited, a non-randomized approach is effective for studying the implementation of evidence-based interventions as part of routine clinical care and may offer rare insights into the efficacy of patient navigation outside of a randomized control trial [47, 48]. In particular, this approach allows for observation and documentation of the real-world variability in fidelity to the multicomponent patient navigation strategy and evidence-based chemotherapy for KS. Thus, it is likely to provide a more accurate evaluation of the impact of patient navigation on real-world outcomes including fidelity to evidence-based chemotherapy and mortality in people with KS.

This study will provide evidence for a multicomponent patient navigation strategy to improve cancer treatment initiation and completion that may be generalizable to other low-resource contexts, including in sub-Saharan Africa. This multicomponent patient navigation strategy, which was developed using a rigorous structured stepwise process, Intervention Mapping, is designed to address common barriers to cancer care in low-resource contexts around the world. This implementation strategy could be adapted to improve cancer care in other low-resource contexts experiencing similar barriers to cancer care. Our implementation science-based approach to evaluation could also be adapted to inform the evaluation of implementation strategies to improve cancer care, which are implemented as part of routine clinical care.

In summary, this type III hybrid effectiveness-implementation evaluation will provide valuable insights into the real-world implementation and impact of a multicomponent patient navigation strategy to improve chemotherapy service penetration and fidelity for people with advanced-stage KS.

## Supplementary Information

**Additional file 1.** StaRI 2017, Standards for Reporting Implementation Studies Checklist.

## Data Availability

Not applicable.

## Abbreviations

KS: Kaposi’s sarcoma
AMPATH: Academic Model Providing Access to Health
ACTG: AIDS Clinical Trials Group Oncology Committee
COM-B: Capability, Opportunity, and Motivation Model of Behavior
AIM: Acceptability of Intervention Measure
IAM: Intervention Appropriateness Measure
FIM: Feasibility of Intervention Measure
TDF: Theoretical Domains Framework
